# Biosynthetic Mechanisms and Biological Significance of Glycerol Phosphate-Containing Glycan in Mammals

**DOI:** 10.3390/molecules26216675

**Published:** 2021-11-04

**Authors:** Rieko Imae, Hiroshi Manya, Tamao Endo

**Affiliations:** Molecular Glycobiology, Research Team for Mechanism of Aging, Tokyo Metropolitan Geriatric Hospital and Institute of Gerontology, 35-2 Sakae-cho, Itabashi-ku, Tokyo 173-0015, Japan; rimae@tmig.or.jp (R.I.); endo@tmig.or.jp (T.E.)

**Keywords:** glycerol phosphate (GroP), α-dystroglycan (α-DG), core M3-type glycan, CDP-glycerol (CDP-Gro), cytidylyltransferase

## Abstract

Bacteria contain glycerol phosphate (GroP)-containing glycans, which are important constituents of cell-surface glycopolymers such as the teichoic acids of Gram-positive bacterial cell walls. These glycopolymers comprising GroP play crucial roles in bacterial physiology and virulence. Recently, the first identification of a GroP-containing glycan in mammals was reported as a variant form of *O*-mannosyl glycan on α-dystroglycan (α-DG). However, the biological significance of such GroP modification remains largely unknown. In this review, we provide an overview of this new discovery of GroP-containing glycan in mammals and then outline the recent progress in elucidating the biosynthetic mechanisms of GroP-containing glycans on α-DG. In addition, we discuss the potential biological role of GroP modification along with the challenges and prospects for further research. The progress in this newly identified glycan modification will provide insights into the phylogenetic implications of glycan.

## 1. Introduction

Glycerol phosphate (GroP) is an important building block of glycopolymers on the bacterial cell surface such as teichoic acids on the cell wall of Gram-positive bacteria [1,2,3]. These glycopolymers play crucial roles in determining the bacterial cell shape, regulating cell division, protection from host defenses, antimicrobial resistance, and pathogenicity [4]. There are three known stereoisomers of GroP, *sn*-glycerol-1-phosphate (Gro1P), *sn*-glycerol-2-phosphate (Gro2P), and *sn*-glycerol-3-phosphate (Gro3P), which are all constituents of bacterial surface glycopolymers. For example, teichoic acids are divided into two types: (i) peptidoglycan-linked glycopolymers (wall teichoic acids), some of which contain phosphodiester polymers composed of Gro3P, and (ii) membrane glycolipid-linked glycopolymers (lipoteichoic acids), which typically contain polymers of Gro1P [5,6]. GroP was not considered to be a glycan constituent of mammalian cells until recently. In 2016, a GroP-containing glycan was identified as a variant form of *O*-mannosyl glycan from a truncated recombinant α-dystroglycan (α-DG) isolated in cultured human cells [7]. However, the biological role of GroP modification on α-DG remains largely unknown. Here, we describe the discovery of GroP-containing glycan in mammals and its biosynthesis including production of the GroP donor CDP-glycerol (CDP-Gro). We further provide an overview of research progress in elucidating the biological significance of GroP-modified glycans and remaining questions to resolve.

## 2. Discovery of GroP-Containing Glycan in Mammals

The unique *O*-mannosyl glycan structure of α-DG containing ribitol-5-phosphate (Rbo5P) was recently revealed [8]. Subsequently, a GroP-modified glycoform was discovered as a variant form of *O*-mannosyl glycan on α-DG rather than Rbo5P [7]. At present, α-DG is the only known carrier protein with GroP modification. In this section, we first describe the biosynthetic mechanism of the Rbo5P-containing *O*-mannosyl glycan of α-DG and then outline the steps leading to the recent discovery of the novel GroP-containing glycoform in mammals.

### 2.1. Overview of the Functional Laminin-Binding Glycan on α-DG

**Figure 1:**
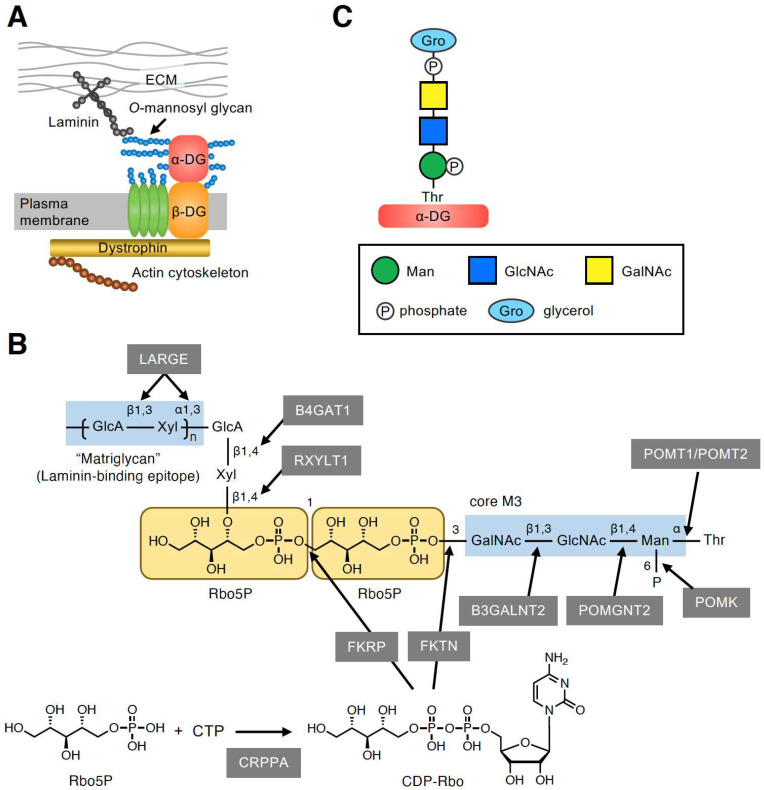
Core M3-type glycan on α-DG. (**A**) The plasma membrane-localized protein complex DGC connects the ECM and actin cytoskeleton. The characteristic *O*-mannosyl glycan on α-DG, namely, the core M3-type glycan, is responsible for binding to some ECM proteins such as laminin. (**B**) Structure of the functional laminin-binding core M3-type glycan and the enzymes involved in its biosynthesis. (**C**) GroP-containing core M3-type glycan structure identified from the glycan analysis of small truncated recombinant α-DG in HEK293T and HCT116 cells [7]. The symbol legends are indicated in the lower box.

The dystrophin–glycoprotein complex (DGC) is a multimeric protein complex located at the plasma membrane (Figure 1A) [9]. The key components of the DGC (i.e., α-DG and β-DG) are encoded by a single gene, DAG1, and are post-translationally cleaved into two subunits that have a non-covalent association [10]. The cell surface-located α-DG is heavily glycosylated and binds to several extracellular matrix (ECM) proteins, such as laminin, via its *O*-mannosyl glycans [11,12,13]. By contrast, the transmembrane protein β-DG interacts intracellularly with dystrophin, which associates with the actin cytoskeleton. Thus, the DGC plays an important role in linking the ECM to the actin cytoskeleton. α-DG is modified by many *O*-linked glycans including mucin-type glycans and *O*-mannosyl glycans in the mucin-like domain [14,15,16]. *O*-mannosyl glycans are classified into three core structures based on the linkage of GlcNAc to the *O*-Man residue: core M1 (GlcNAcβ1-2Man), core M2 [GlcNAcβ1-2(GlcNAcβ1-6)Man], and core M3 (GalNAcβ1-3GlcNAcβ1-4Man). At present, core M3-type glycan has only been identified on α-DG, and the laminin-binding epitope, which is a repeating unit of GlcA-Xyl, was proved to be formed on the core M3-type glycan [17]. However, the complete glycan structure has not yet been elucidated owing to technical difficulties related to its large size and unknown negative charges. Recently, the precise structure of the functional laminin-binding core M3-type glycan, [(3GlcAβ1–3Xylα1)_n_-3GlcAβ1–4Xylβ1–4Rbo5P-1Rbo5P-3GalNAcβ1–3GlcNAcβ1–4(phospho-6)Manα1], was revealed by several groups, including our own (Figure 1B) [8,18,19,20,21]. This was also the first demonstration of the involvement of Rbo5P, a pentose alcohol phosphate known as a constituent of bacterial surface glycopolymers [5], in mammalian glycans.

The enzymes involved in the biosynthesis of functional core M3-type glycan have also been identified. The initial Man residue common to the three core structures of *O*-mannosyl glycans is transferred to the many Ser/Thr residues of α-DG by the protein *O*-mannosyltransferases POMT1/POMT2 in the endoplasmic reticulum (ER) (Figure 1B) [22]. The core M3 structure is then synthesized at specific sites, such as Thr317, Thr319, and Thr379, on human α-DG [7,17,18,23,24] with the addition of GlcNAc and GalNAc by protein *O*-mannose *N*-acetylglucosaminyltransferase 2 (POMGNT2) and β1,3-*N*-acetylgalactosaminyltransferase 2 (B3GALNT2), respectively [25]. After the phosphorylation of Man at the C6 position by protein *O*-mannose kinase (POMK) [25], α-DG is transported to the Golgi, and the first Rbo5P is transferred by fukutin (FKTN) from the activated precursor CDP-ribitol (CDP-Rbo) to the C3 position of GalNAc. The second Rbo5P is then transferred by fukutin-related protein (FKRP) from CDP-Rbo to the C1 position of the first Rbo5P to form a tandem Rbo5P structure [8]. CDP-Rbo is synthesized from Rbo5P and CTP by CDP-L-ribitol pyrophosphorylase A (CRPPA) [8,19]. A GlcAβ1-4Xylβ1-4 unit is then formed by the sequential action of ribitol xylosyltransferase 1 (RXYLT1) and β1,4-glucuronyltransferase 1 (B4GAT1) [18,26,27]. Finally, the (-3GlcAβ1-3Xylα1-) repeating unit, which is the laminin-binding epitope known as matriglycan, is formed by like-acetylglucosaminyltransferase (LARGE) [28]. The term matriglycan was derived from the fact that α-DG functions as a matrix receptor and this glycan structure is the binding epitope for matrix proteins [29]. The link between laminin and α-DG plays critical roles in sarcolemmal stability and neural cell migration in the developing brain; accordingly, defects in the biosynthesis of core M3-type glycan cause a group of congenital muscular dystrophies with brain malformation collectively termed α-dystroglycanopathies [11,12,13]. 

### 2.2. Discovery of the GroP-Containing Glycoform of α-DG

Yagi et al. [7] designed and expressed a small truncated recombinant α-DG protein in cultured human cells. The purified protein was subjected to liquid chromatography (LC)-tandem mass spectrometry (MS/MS) analysis, which identified structures corresponding to the Rbo5P-containing core M3-type glycan, confirming our findings [8]. Interestingly, they also identified the core M3-type glycan which contained a GroP [GroP-(phospho)core M3] (Figure 1C). Although the stereochemistry of GroP had not been elucidated, this was the first report to demonstrate the presence of GroP-containing glycans in mammals. Notably, GroP was found to be modified at the non-reducing terminus of phospho-core M3 instead of the characteristic RboP modification; however, no glycoform with further modification of GroP was observed. In the human embryonic kidney cell line, HEK293T, the glycoforms with RboP modification appeared to be more abundant than those with GroP modification, whereas the abundance of these two glycoforms seemed to be comparable in the human colon cancer cell line HCT116 [7]. In contrast, the GroP-modified glycoform was not detected in our glycan analysis of α-DG expressed in NIH 3T3 cells, a murine fibroblast cell line [8]. This suggests that the occurrence of GroP modification may vary depending on the cell type.

## 3. Mechanism of GroP Transfer on α-DG Glycan

**Figure 2:**
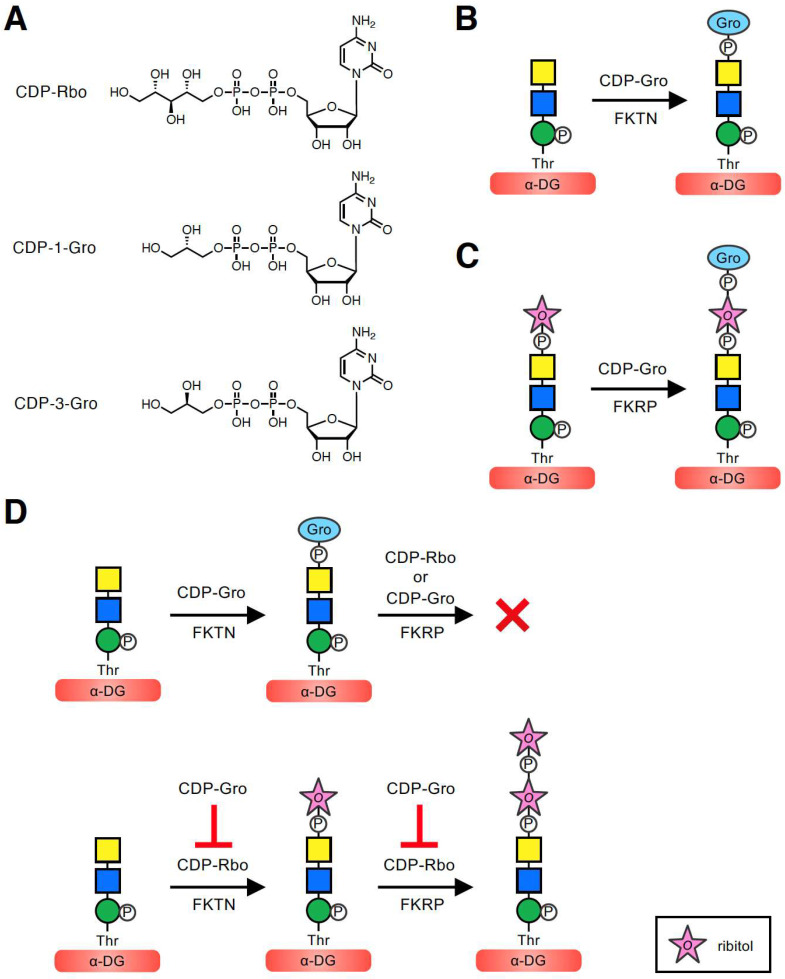
GroP transfer activities of FKTN and FKRP using CDP-Gro and the inhibitory effect of CDP-Gro on elongation of core M3-type glycan. (**A**) Structures of CDP-Rbo and CDP-Gro. The two stereoisomers of CDP-Gro, CDP-1-Gro and CDP-3-Gro, are shown. CDP-Rbo is defined as CDP-5-D-Rbo. CDP-1-Gro and CDP-3-Gro are defined as CDP-1-L-Gro and CDP-3-L-Gro, respectively. (**B**) FKTN transfers GroP from CDP-Gro to the terminal GalNAc of the phospho-core M3 structure. (**C**) FKRP transfers GroP from CDP-Gro to the terminal Rbo5P of the Rbo5P-(phospho)core M3 structure. (**D**) CDP-Gro inhibits elongation of the core M3-type glycan by two mechanisms. (**Upper**) When CDP-Gro serves as the donor substrate for FKTN, the GroP-transferred product does not serve as the acceptor substrate for FKRP, and further extension of the outer glycan chain cannot occur. (**Lower**) CDP-Gro competitively inhibits the Rbo5P transfer reaction from CDP-Rbo of both FKTN and FKRP, also preventing elongation of the outer glycan chain. The symbol legend for ribitol is indicated in the lower box. Other symbol legends are the same as in Figure 1C.

To better understand the implications of these modifications, it is first important to describe the mechanism by which GroP is transferred to the core M3-type glycan. In the biosynthesis of bacterial wall teichoic acids, tagB and tagF transfer Gro3P using CDP-Gro (more specifically, CDP-3-Gro) as the activated precursor [1]. In addition, in the biosynthesis of lipoteichoic acids, ltaS transfers Gro1P from the membrane phospholipid phosphatidylglycerol [5]. However, there are no human homologs of these enzymes. Moreover, when Yagi et al. [7] analyzed the glycoform of recombinant α-DG expressed in HCT116 cells deficient in FKTN, no GroP-containing as well as RboP-containing glycoforms were found, implying that FKTN might be involved in transferring not only RboP but also GroP to the phospho-core M3 structure. However, it was unclear whether FKTN itself transfers GroP, serves as a co-factor of an unknown GroP transferase, or plays a role in the transfer via another mechanism. To address these questions, we examined whether FKTN has GroP transferase activity in vitro [30]. Because FKTN transfers Rbo5P using CDP-Rbo as a donor substrate, we assumed that the GroP transfer reaction would involve a similar catalytic mechanism using CDP-Gro. Because the pure CDP-Gro stereoisomer was not commercially available, we used mix-CDP-Gro, a mixture of two CDP-Gro stereoisomers, CDP-1-Gro and CDP-3-Gro, which serve as the donors of Gro1P and Gro3P, respectively (Figure 2A). When purified recombinant FKTN was incubated with mix-CDP-Gro and a phospho-core M3-modified peptide, the GroP-transferred product was revealed. FKTN can transfer GroP to the terminal GalNAc of the phospho-core M3 structure, consistent with the GroP-containing glycoform identified on recombinant α-DG (Figure 1C) [7]. These results indicated that FKTN is a GroP transferase that uses CDP-Gro as a donor (Figure 2B). 

Next, to determine which stereoisomer, CDP-1-Gro or CDP-3-Gro, serves as the donor substrate for FKTN, we enzymatically synthesized CDP-1-Gro and CDP-3-Gro using Gro1P and Gro3P, respectively, with the bacterial glycerol-phosphate cytidylyltransferase (GCT) [31]. We then conducted a GroP-transfer assay of FKTN using CDP-1-Gro or CDP-3-Gro, which showed that both isomers can serve as donor substrates. Thus, it was revealed that FKTN can use three donor substrates: CDP-Rbo, CDP-1-Gro, and CDP-3-Gro. Kinetic analysis was further performed to compare the efficiency of these compounds as donors for FKTN [30]. The *K_m_* values of FKTN for these three donors were similar, suggesting a comparable affinity for FKTN. In other words, the modifications of glycerol and ribitol did not substantially affect the affinity of the substrate for FKTN. However, the *V*_max_ value for CDP-Rbo was significantly higher than those for CDP-1-Gro and CDP-3-Gro, suggesting that the larger size of ribitol than glycerol contributes to more efficient transfer. In addition, the relative catalytic efficiency (*V*_max_/*K_m_*) values for CDP-Rbo, CDP-1-Gro, and CDP-3-Gro were 0.184, 0.026, and 0.017 h^−1^, respectively, indicating that CDP-Rbo is the best donor substrate for FKTN. In addition, CDP-1-Gro appears to be a slightly more preferred substrate for FKTN than CDP-3-Gro. This may be because the orientation of the hydroxyl group at the β-carbon of the phosphate is the same in CDP-Rbo and CDP-1-Gro, which is the preferred orientation of FKTN (Figure 2A). However, because mammalian cells appear to contain CDP-3-Gro but not CDP-1-Gro [32] (see Section 6), it is assumed that FKTN only transfers Gro3P in vivo. 

Because FKRP also exhibits Rbo5P transfer activity using CDP-Rbo, and its catalytic domain shows significant sequence similarity to that of FKTN [33], we considered that FKRP may also have GroP transfer activity using CDP-Gro. When purified FKRP was incubated with mix-CDP-Gro and its acceptor Rbo5P-(phospho)core M3-modified peptide, the GroP-transferred product was obtained. The position where FKRP transfers GroP is considered to be the terminal Rbo5P of the acceptor glycan. These results showed that FKRP can synthesize the GroP-Rbo5P-(phospho)core M3 structure in vitro (Figure 2C) [30]. Similar to FKTN, the GroP transfer activity of FKRP appeared to be lower than its Rbo5P transfer activity.

Recently, we determined the crystal structure of FKRP with the primary donor (CDP-Rbo), and the acceptor recognition mechanisms were elucidated [34]. We found that the catalytic domain of FKRP recognizes CDP-Rbo. In particular, the interactions of four Asp residues in the active site with the phosphate residue of CDP-Rbo appeared to be essential for the enzyme activity, but the Rbo moiety was not recognized definitely. Therefore, when CDP-Gro, which is smaller than CDP-Rbo, interacts with FKRP, it can be easily accommodated at the donor-binding site. Because of the high sequence identity between the catalytic domains of FKTN and FKRP, FKTN may recognize CDP-Rbo and CDP-Gro in a similar manner. In summary, FKTN and FKRP were the first enzymes identified to have GroP transfer activity in mammals.

## 4. Inhibitory Effects of GroP-Modified Glycan and CDP-Gro on the Synthesis of the Laminin-Binding Glycan of α-DG

To determine the biological role of GroP modification, we examined the effect of GroP modification on synthesis of the core M3-type glycan [30]. Specifically, we tested whether the GroP-transferred product of FKTN, the GroP-(phospho)core M3-modified peptide, serves as an acceptor substrate for FKRP (Figure 2D). Purified FKRP was incubated with CDP-Rbo and GroP-(phospho)core M3 peptide, and the products were analyzed. We found that the GroP-(phospho)core M3-modified peptide no longer exerted its function as an acceptor for FKRP. We also tested whether FKRP uses CDP-Gro as a donor substrate to extend the GroP-(phospho)core M3 structure, but no new product was detected. These results demonstrated that the GroP-transferred phospho-core M3 structure cannot be further extended by FKRP with either Rbo5P or GroP in vitro, consistent with the previous finding that any glycoform with further modification of GroP was not observed in the glycoform analysis of recombinant α-DG [7].

In addition, given that the kinetic data of FKTN indicated the comparable affinity of CDP-Rbo and CDP-Gro, we speculated that CDP-Gro may act as a competitive inhibitor of CDP-Rbo. To verify this assumption, we examined the effect of the coexistence of CDP-Rbo and CDP-Gro on the Rbo5P transfer activity of FKTN [30]. For this purpose, we used ^3^H-labeled CDP-Rbo (CDP-[^3^H]Rbo) to monitor the Rbo5P transfer activity, and the competitive effect of CDP-Gro was assessed using different molar ratios of CDP-Rbo to CDP-Gro. We found that the Rbo5P transfer activity of FKTN decreased with an increasing molar ratio of CDP-Gro to CDP-Rbo: the Rbo5P transfer activity decreased to 63% (CDP-Rbo:CDP-Gro, 1:1) and 30% (1:5) of that without CDP-Gro. This result confirmed that CDP-Gro inhibits the Rbo5P transfer activity of FKTN. In addition, the Rbo5P transfer activity of FKTN was not affected by ribitol, Rbo5P, or Gro3P, indicating that the CDP moiety is important for the inhibitory effect of CDP-Gro. We also found that the Rbo5P transfer activity of FKRP to the Rbo5P-(phospho)core M3 peptide decreased with an increasing molar ratio of CDP-Gro to CDP-Rbo: the decrease of Rbo5P transfer activity to 65% (CDP-Rbo:CDP-Gro, 1:1) and 19% (1:5) of that without CDP-Gro. These results indicated that CDP-Gro inhibited the Rbo5P transfer activity of FKRP and FKTN in a concentration-dependent manner.

Collectively, these experiments showed that CDP-Gro has inhibitory effects on the synthesis of the functional laminin-binding glycan of α-DG in vitro by two mechanisms (Figure 2D). First, the GroP-transferred product by FKTN does not serve as the acceptor substrate for FKRP, and further extension of the outer glycan chain cannot proceed. Because FKRP transfers RboP to the C1 position of the first RboP transferred by FKTN (Figure 1B) [8], the peripheral GroP may not be long enough for the catalytic active site of FKRP. Second, since CDP-Gro competitively inhibits the RboP transfer reaction of both FKTN and FKRP with CDP-Rbo as the substrate, elongation of the outer glycan chain is prevented. However, the effect of GroP modification by FKRP on core M3-type glycan synthesis has not been determined to date.

## 5. Detection of CDP-Gro in Mammals

Microorganisms, such as bacteria and archaea, are well known to synthesize CDP-Gro. However, it was unknown whether mammalian cells contain CDP-Gro. To explore the presence of CDP-Gro in mammals, we developed a quantitative LC-MS/MS method specific to CDP-Gro analysis [32]. Because GroP-modified glycoforms were detected in HEK293T and HCT116 cells [7], it seemed likely that these cells contain CDP-Gro. Therefore, we examined three human cultured cell lines, HEK293T, HCT116, and HAP1 cells, which were all confirmed to contain CDP-Gro. To our knowledge, this was the first report of the presence of CDP-Gro in mammals. We also measured the CDP-Rbo content in the cells and found that the ratio of CDP-Gro to CDP-Rbo varied depending on the cell type: in HEK293T cells, the CDP-Gro content was less than 10% that of CDP-Rbo, whereas in HCT116 and HAP1 cells, the content of CDP-Gro and CDP-Rbo was comparable. Considering the competitive action of CDP-Gro and CDP-Rbo, it is expected that GroP modification is more likely to occur when the CDP-Gro to CDP-Rbo ratio is high. Indeed, the CDP-Gro to CDP-Rbo ratio in the cells was well correlated with the previous observation: RboP-modified glycans appeared to be predominant in HEK293T cells, whereas the abundance of RboP- and GroP-modified glycans seemed to be comparable in HCT116 cells [7]. 

To further determine whether mammalian tissues contain CDP-Gro, we examined various mouse tissues (brain, liver, heart, skeletal muscle, kidney, and lung) [32]. All of the tissues examined contained CDP-Gro, but the content was lower than that of CDP-Rbo in most tissues. Exceptionally, in the liver, the ratio of CDP-Gro to CDP-Rbo was relatively high: the CDP-Gro content was 41% of that of CDP-Rbo. This suggested that GroP modification tends to occur in the liver. Determining the actual ratio of RboP- and GroP-modified glycans on α-DG and the biological role of GroP-containing glycan in the liver will be interesting subjects for future study.

## 6. Biosynthetic Mechanism of CDP-Gro in Mammalian Cells

**Figure 3:**
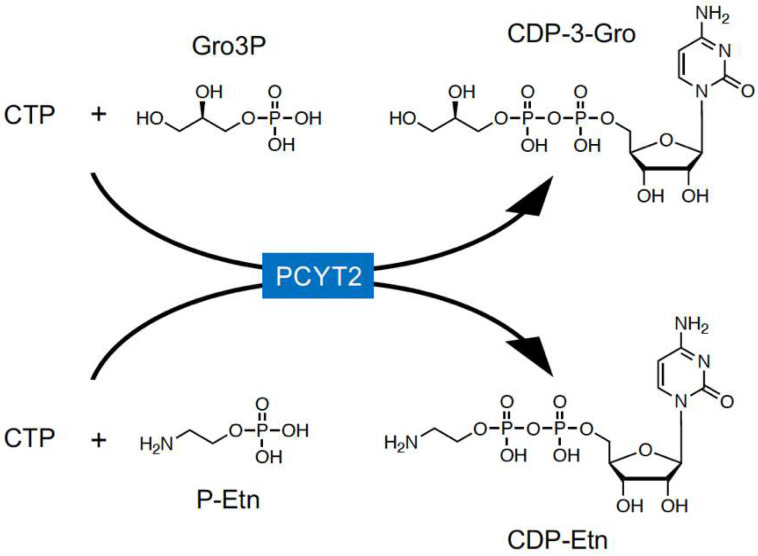
In mammals, PCYT2 catalyzes the synthesis of CDP-Etn from CTP and P-Etn, and PCYT2 also synthesizes CDP-3-Gro from CTP and Gro3P.

In mammals, CDP-Rbo is synthesized from RboP and CTP by CRPPA (Figure 1B) [8,19]. However, CRPPA does not have CDP-Gro synthetic activity [35], and GroP modification was not reduced in CRPPA-deficient cells [7], indicating that mammals likely have other enzyme(s) to catalyze CDP-Gro synthesis. In microorganisms, CDP-Gro is known to be synthesized from GroP and CTP by the action of GCT; however, this enzyme is not conserved in mammals. GCT belongs to a family of cytidylyltransferase, and mammals have three members of the family: PCYT1A, PCYT1B, and PCYT2 [36,37]. PCYT1A and PCYT1B are choline-phosphate cytidylyltransferases that synthesize CDP-choline from phosphocholine and CTP [38,39], and PCYT2 is an ethanolamine-phosphate cytidylyltransferase that synthesizes CDP-ethanolamine (CDP-Etn) from phosphoethanolamine (P-Etn) and CTP [40], the key regulatory enzymes in the biosynthetic pathway of phosphatidylcholine (PC) and phosphatidylethanolamine (PE), respectively. Therefore, we considered that these enzymes may also have CDP-Gro synthetic activity. In mammalian cells, Gro3P is synthesized from dihydroxyacetone phosphate (DHAP) by Gro3P dehydrogenase (GPDH) or via glycerol phosphorylation by glycerol kinase [41], but Gro1P is not synthesized at all. Therefore, it was assumed that mammalian cells synthesize CDP-3-Gro from Gro3P and CTP. We found that CDP-Gro synthetic activity from Gro3P and CTP was significantly increased in the lysate of PCYT2-overexpressing cells, but not in that of PCYT1A- or PCYT1B-overexpressing cells. Furthermore, there are two major splicing isoforms of PCYT2 in humans (PCYT2α and -β) [40,42,43], which showed almost identical CDP-Gro synthetic activities in our study [32]. In addition, reduced expression of PCYT2 in HAP1 and HCT116 cells prominently decreased the CDP-Gro content by approximately 90%, indicating the major contribution of PCYT2 in CDP-Gro synthesis. As expected, the CDP-Etn content also decreased, although this decrease was relatively mild. These results collectively indicated that PCYT2 functions as a CDP-Gro synthase in human cells (Figure 3) [32]. Because the liver shows high glycerol kinase activity [41], the large amount of Gro3P may explain the high CDP-Gro content found in the liver [32]. Our data also indicated that PCYT2 has two different enzyme activities using P-Etn and Gro3P, playing dual roles in the biosynthesis of phospholipids and glycans, respectively. Because P-Etn and Gro3P have similar molecular sizes, the PCYT2 active site may be able to accommodate both substrates. Nevertheless, structural determination of PCYT2 with P-Etn and Gro3P is needed to further understand the detailed substrate recognition mechanism of PCYT2.

PE is the second most abundant phospholipid in mammals and plays important roles in various cellular processes such as membrane fusion and autophagy [44,45]. PE is biosynthesized by two pathways: the CDP-Etn pathway (also known as the Kennedy pathway) in the ER and the decarboxylation of phosphatidylserine in the mitochondria. The CDP-Etn pathway is the major route for PE production via condensation of CDP-Etn and diacylglycerol. PCYT2 is the rate-limiting enzyme in the CDP-Etn pathway. Complete loss of PCYT2 in mice causes embryonic lethality, indicating its importance in mammalian development [46]. PCYT2 heterozygous mice developed adult-onset metabolic disease phenotypes, such as hyperlipidemia, obesity, and insulin resistance [47], and showed male-specific cardiac dysfunction [48]. In addition, the involvement of PCYT2 in cancer [49,50,51,52], herpesvirus replication and pathogenicity [53], and complex hereditary spastic paraplegia [54] has been reported. Thus, PCYT2 is involved in various physiological and pathological processes. In addition to PE synthesis, changes in GroP-containing glycan synthesis may also be involved in these events. Since PCYT2 is known to be phosphorylated by protein kinase C or other kinases [43], the phosphorylation level of PCYT2 may regulate its role in the synthesis of CDP-Etn and CDP-Gro.

Because PCYT2 is mainly localized in the cytosol [55], CDP-Gro is produced in the cytosol and needs to be transported into the lumen of the Golgi where the GroP transfer reaction occurs by FKTN or FKRP. However, the specific CDP-Gro transporter has not yet been identified. Recently, the Golgi-localized proteins SLC35A1 and SLC35A4 were shown to have redundant functions in the transport of CDP-Rbo into the Golgi [56]. SLC35A1 is a CMP-sialic acid transporter [57] with a large substrate-binding pocket to accommodate the bulky CMP-sialic acid. However, other cytidine phosphate-coupled molecules, such as CDP-Rbo, which is smaller than CMP-sialic acid, may also be transported by SLC35A1. In contrast, SLC35A4 is an orphan nucleotide sugar transporter, but it harbors the conserved nucleotide-interacting residues of SLC35A1 [58]. Interestingly, SLC35A4 has a relatively smaller substrate-binding pocket than that of SLC35A1, indicating that it may accommodate CDP-Rbo but not bulky CMP-sialic acid, suggesting that SLC35A4 may be a CDP-Rbo transporter. Taken together, these findings suggest that CDP-Gro, which is smaller than CDP-Rbo, may be accommodated in the substrate-binding pockets of both SLC35A1 and SLC35A4, which can then transport CDP-Gro to the lumen of the Golgi.

## 7. Biological Roles of GroP-Containing Glycan on α-DG

Although we confirmed that GroP modification of the core M3-type glycan by FKTN inhibits functional glycan synthesis on α-DG in vitro [30], the biological roles of GroP-modified glycan remained unclear. To elucidate this issue, we examined the effect of the decreased CDP-Gro content on the α-DG glycan by reducing PCYT2 expression [32]. When PCYT2 expression was reduced in HAP1 cells, the cellular contents of CDP-Gro and CDP-Etn were reduced by approximately 95% and 40–70%, respectively. Under these conditions, Western blot analysis showed that reactivity of IIH6 antibody, which recognizes the laminin-binding epitope (matriglycan) on α-DG, increased and a slightly higher-molecular-weight band was observed, suggesting that the functional core M3-type glycan synthesis is increased by PCYT2 suppression. This result is consistent with the inhibitory effect of GroP modification on functional core M3-type glycan synthesis found in vitro [30]. Interestingly, we also found that the expression of α-DG protein itself was increased by PCYT2 suppression. This may not be due to the increased *DAG1* gene expression but rather via post-transcriptional regulation of α-DG protein levels. This suggests that GroP modification of α-DG may reduce the stability of α-DG protein. We also found that the laminin-binding activity of α-DG was markedly increased by PCYT2 reduction. Collectively, these results suggest that GroP-containing glycan inhibits the function of α-DG by inhibiting functional glycan synthesis and reducing the expression of α-DG. However, PCYT2 suppression also reduced the CDP-Etn content, albeit to a relatively mild degree. As described above, because PE is mainly synthesized by the CDP-Etn pathway, the PE content may also be reduced to some extent by PCYT2 reduction. Currently, the possibility that the reduction of PE content in the membrane affects the biochemical properties of α-DG cannot be excluded. Therefore, further careful investigation of the function of GroP modification in cells is needed. If there is a critical amino acid of PCYT2 that affects specifically either CDP-Etn or CDP-Gro synthetic activity, it may be useful to distinguish between the CDP-Etn- and CDP-Gro-related functions of PCYT2. Very recently, Yamasaki et al. [59] showed that overexpression of bacterial GCT in HCT116 cells significantly increased cellular CDP-Gro, and GroP modification of α-DG increased and laminin-binding glycan largely decreased. These results suggest the inhibitory role of GroP modification in the functional glycan synthesis of α-DG at the cellular level.

The laminin-binding glycan of α-DG plays a critical role in maintaining muscle integrity and neuronal migration in the developing brain [60]. α-DG is also expressed in various other tissues [61], and its apparent molecular mass is tissue-specific due to the differences in glycosylation [62,63]. In addition, the laminin-binding glycan of α-DG is frequently lost in many cancer tissues and cells [64,65,66,67,68,69], which may result in the reduction of cell–ECM interaction and cell polarity, causing cancer progression and metastasis [67,68,70]. The lack of functional glycan of α-DG in cancer is explained by reduced expression of the glycosyltransferase genes for core M3-type glycan synthesis in some cases [67,68,71,72,73,74]. Alternatively, GroP modification may play an important role in fine-tuning the strength of the binding between the cell and ECM in normal tissues or cancers. Thus, it is important to reveal the mechanism underlying regulation of functional α-DG expression besides directly changing the core M3-type glycan synthetic glycosyltransferases. Some mechanistic insight can be suggested by the reported influence of human natural killer-1 sulfotransferase (HNK-1ST) on formation of the laminin-binding glycan on α-DG by competing with LARGE [75,76], suggesting that sulfation may act as a stop signal of glycan elongation, similar to GroP modification. In addition, we propose a novel mechanism by which GroP modification regulates the formation of functional glycan on α-DG and α-DG expression, depending on the ratio of cellular CDP-Rbo to CDP-Gro.

## 8. Prospects

Because GroP modification of α-DG is considered as a stop signal of functional glycan synthesis, it may be involved in the pathogenesis of α-dystroglycanopathies or cancer progression. Yamasaki et al. [59] established an antibody that is highly reactive with GroP-terminated glycan. This antibody may be useful to elucidate the tissue distribution and the role of GroP-modification under the physiological and pathological conditions. In addition to the regulatory role of glycan extension, the possibility of a functional role of GroP modification itself should be considered such as a new ligand in cellular communication. Identification of the mammalian CDP-Gro synthesizing enzyme PCYT2 is expected to further promote research toward the elucidation of the functional role of GroP-containing glycans in the future. However, because PCYT2 also plays an important role in PE synthesis, strategies are needed to ensure only manipulating GroP modification to best uncover the specific physiological function of this modification. Gro3P is derived from reduction of the glycolytic intermediate DHAP, and it is also the initial substrate for de novo glycerolipid biosynthesis [41]. Thus, Gro3P is an important metabolic intermediate linking glucose and lipid metabolism. For example, the change in Gro3P content is highly associated with triacylglycerol levels in the liver [77,78,79,80]. Gro3P content fluctuations may affect the occurrence of GroP-containing glycans. Therefore, it is important to further explore the role of GroP-containing glycans in the context of cell metabolism. 

Moreover, it will be important to assess whether any molecules (proteins or lipids) other than α-DG have GroP-containing glycans in mammals. If other molecules can serve as the acceptor substrate for FKTN or FKRP, GroP-modified molecules other than α-DG would be present. Another possibility is the presence of Gro1P-containing glycans. Since mammals appear to contain CDP-3-Gro but not CDP-1-Gro [32], only Gro3P-modified glycan is assumed to be produced using CDP-Gro. However, it is possible that a Gro1P-containing glycan derived from the head group of the minor membrane phospholipid phosphatidylglycerol exists in mammals [5], similar to bacteria. Further advances in glycan analysis techniques may contribute to the identification of new molecules with GroP modification. These findings will in turn promote our understanding of GroP-containing glycans in mammals, toward ultimately gaining insight into the phylogenetic relationships of glycan on the cell surface and their evolution from bacteria to mammals.

## Data Availability

Not applicable.
